# A Machine Learning Approach to Predicting Diabetes Complications

**DOI:** 10.3390/healthcare9121712

**Published:** 2021-12-09

**Authors:** Yazan Jian, Michel Pasquier, Assim Sagahyroon, Fadi Aloul

**Affiliations:** Department of Computer Science and Engineering, American University of Sharjah, Sharjah 26666, United Arab Emirates; mpasquier@aus.edu (M.P.); asagahyroon@aus.edu (A.S.); faloul@aus.edu (F.A.)

**Keywords:** diabetes prediction, diabetes complications, supervised learning

## Abstract

Diabetes mellitus (DM) is a chronic disease that is considered to be life-threatening. It can affect any part of the body over time, resulting in serious complications such as nephropathy, neuropathy, and retinopathy. In this work, several supervised classification algorithms were applied for building different models to predict and classify eight diabetes complications. The complications include metabolic syndrome, dyslipidemia, neuropathy, nephropathy, diabetic foot, hypertension, obesity, and retinopathy. For this study, a dataset collected by the Rashid Center for Diabetes and Research (RCDR) located in Ajman, UAE, was utilized. The dataset consists of 884 records with 79 features. Some essential preprocessing steps were applied to handle the missing values and unbalanced data problems. Furthermore, feature selection was performed to select the top five and ten features for each complication. The final number of records used to train and build the binary classifiers for each complication was as follows: 428—metabolic syndrome, 836—dyslipidemia, 223—neuropathy, 233—nephropathy, 240—diabetic foot, 586—hypertension, 498—obesity, 228—retinopathy. Repeated stratified k-fold cross-validation (with k = 10 and a total of 10 repetitions) was employed for a better estimation of the performance. Accuracy and F1-score were used to evaluate the models’ performance reaching a maximum of 97.8% and 97.7% for accuracy and F1-scores, respectively. Moreover, by comparing the performance achieved using different attributes’ sets, it was found that by using a selected number of features, we can still build adequate classifiers.

## 1. Introduction

Diabetes mellitus, or diabetes for short, is a chronic disease that occurs either when the pancreas does not produce enough insulin or when the body cannot effectively use the insulin it produces [1]. Diabetes has two main types called type 1 and type 2. In type 1 diabetes (also known as insulin-dependent or childhood-onset), there is insulin production deficiency in the body, which requires daily administration of insulin, whereas in type 2 diabetes (known formally as non-insulin-dependent or adult-onset), the body cannot use insulin effectively. According to the World Health Organization (WHO), the number of people with diabetes in 2014 was 422 million. Moreover, in 2016, diabetes was the direct cause of 1.6 million deaths [1].

There are different causes for diabetes. For instance, type 1 diabetes mellitus (T1DM) can develop due to an autoimmune reaction that destroys the cells in the pancreas that make insulin, called beta cells [2], whereas type 2 diabetes is mainly caused by age, family history of diabetes, high blood pressure, high levels of triglycerides, heart disease or stroke [3]. Early detection of diabetes can be of great benefit, especially because the progression of prediabetes to type 2 diabetes is quite high. According to CDC [4], diabetes can affect any part of the body over time, leading to different types of complications. The most common types are divided into micro- and macrovascular disorders. The former are those long-term complications that affect small blood vessels, including retinopathy, nephropathy, and neuropathy. Macrovascular disorders, however, include ischemic heart disease, peripheral vascular disease, and cerebrovascular disease [5].

Due to high diabetes mortality and morbidity along with its possible complications, it is very important to understand how to deal with diabetes and how to prevent such possible complications.

To reduce the possibility of developing some serious complications related to diabetes, machine learning and data mining techniques can be applied to diabetes-related datasets. Machine learning is a branch of artificial intelligence and computer science which focuses on the use of data and algorithms to imitate the way that humans learn. Machine learning itself can be divided into two main categories, namely, supervised and unsupervised learning [6]. The main goal in both cases is to make use of a given dataset to enhance our understanding of the data and discover useful knowledge. Supervised machine learning is characterized by the use of labeled data to train its algorithms and can be utilized for classification or regression tasks. The goal of classification is to assign each unknown instance to one of possible classes or categories for prediction or diagnosis purposes.

The proposed work implements several supervised machine learning techniques and algorithms to predict different complications related to diabetes. Unlike typical diabetes datasets, the complications’ set consists of various collections of complications such as metabolic syndrome, dyslipidemia, neuropathy, nephropathy, diabetic foot, hypertension, obesity, and retinopathy. Furthermore, logistic regression (LR), support vector machine (SVM), decision tree (DT CART), random forest (RF), AdaBoost, and XGBoost were utilized to build and evaluate different resulting classifiers. The contributions of this work are as follows:

- Implementation and evaluation of traditional and ensemble machine learning models to predict eight complications in diabetic patients by utilizing a comprehensive UAE-based dataset.
- Identification of the dominant characteristics that may lead to diabetic complications using feature selection methods.

## 2. Literature Review

Data mining can be utilized in different sectors such as education, healthcare, business, and many other fields. The applications of data mining in healthcare enable disease diagnosis, prognosis, and a deep understanding of medical data [7,8]. For instance, it may provide a better understanding of the correlation between different chronic diseases [6], such as diabetes mellitus (DM), which is a serious health problem and a cause of death.

Diagnosis and prognosis of DM have received a lot of attention. Hasan et al. [9] proposed a new approach for diabetes prediction using the PIMA Indians Diabetes (PIDD) dataset. The dataset consists of 768 female patients, specifically, 268 diabetic patients (positive) and 500 non-diabetic patients (negative) with eight different attributes: pregnant, glucose, pressure, triceps, insulin, BMI, pedigree, and age. As mentioned by the authors, preprocessing is the heart of achieving state-of-the-art results, which consists of outlier rejection, substitution with the mean for missing values, data standardization, feature selection, and k-fold cross-validation (fivefold in this case). Decision trees, *k*-NN, AdaBoost, random forest, naïve Bayes, and XGBoost were all used and tested in this study. The authors also used an ensemble technique that aimed to boost the performance using a group of classifiers. In ensemble methods, the aggregation of outputs from different models can improve precision of the prediction. The best models used together were AdaBoost and XGBoost. The area under the curve (AUC) was chosen as the performance metric. The paper was able to achieve an AUC score of 0.95 which outperformed other studies.

Sisodia et al. [10] aimed to prognosticate the likelihood of diabetes in patients with maximum accuracy. The PIDD dataset used in this paper is the same as the previous one [9]. A decision tree, SVM, and naïve Bayes were all used in this experiment to detect diabetes at an early stage. Accuracy, precision, and recall as well as the F-score were used to measure the best model performance. As reported in the paper, naïve Bayes achieved the best performance results, with a maximum accuracy of 76.3%.

In [11], a performance comparison between three data mining models for predicting diabetes or prediabetes was discussed. The data mining models were logistic regression (LR), artificial neural network (ANNs), and decision trees (DT). The balanced dataset used consists of 735 patients and 752 normal controls. The 12 attributes used in building the models were gender, age, marital status, educational level, family history of diabetes, BMI, coffee drinking, physical activity, sleep duration, work stress, consumption of fish, and preference for salty foods. All the previous attributes were gathered by means of a questionnaire. The authors concluded that the C5.0 decision tree performed the best for classification accuracy.

Abdulhadi et al. [12] constructed several machine learning models to predict the presence of diabetes in women using the PIDD dataset. The authors addressed the missing values problem by using the mean substitution method and rescaled all the attributes using a standardization method. LR, linear discriminant analysis (LDA), SVM (linear and polynomial), RF were used to build the models. According to the paper, a maximum accuracy score of 82% was achieved by the RF model.

In addition to predicting the presence of diabetes in patients, few existing studies have reported the use of machine learning to develop prediction models of diabetes complications. For instance, in [13], a model was built to predict some chronic diabetes complications, especially eye disease, kidney disease, coronary heart disease, and hyperlipidemia. The authors started with a dataset of 455 records. The number of records decreased through data selection and cleaning. The final number of records as well as the number of features used to build the model were not mentioned in the paper. The authors used an iterative decision tree (ID3) algorithm to build the model [14]. To evaluate its performance, 10-fold cross validation was used, yielding an accuracy of 92.35%. It is worth mentioning that the high accuracy score in this case is not sufficient to indicate the performance of the model, especially in case of unbalanced data. This is mainly because a model can ignore the minority class by predicting all the instances as the majority class and still achieve good accuracy scores.

In [15], HbA1c regression models were developed. As mentioned in the paper, HbA1c reflects the average amount of glucose accumulated in the blood over the last 2–3 months and has direct relationships with diabetes and future risk of complications. The dataset used in this study was collected from the Diabetes Research in Children Network (DirecNet) trials on 170 subjects having type 1 diabetes mellitus and aged between 4 to <10 years. The missing data problem was addressed using the mean substitution while any attribute with missing values >20% was discarded. Moreover, several feature extraction and selection methods were applied to the dataset. According to the paper, the final ML model which consisted of two ensemble methods RF and extreme gradient boosting (XGB) achieved a low mean absolute error (MAE) of 3.39 mmol/mol and a high coefficient of determination (R-squared) score of 0.81.

Dagliati et al. [16] focused on predicting the onset of retinopathy, neuropathy, and nephropathy in T2DM patients in different time scenarios, at 3, 5, and 7 years from the first visit to the hospital. The first visit to the hospital provided the patient’s health status. The selection of patients in this study consisted of the following criteria: patient has a follow-up time longer than the corresponding temporal threshold (3, 5, or 7); patient develops the complication after the first visit; patient’s complication onset date has been registered. The dataset was collected by Istituto Clinico Scientifico Maugeri (ICSM), Hospital of Pavia, Italy, for over 10 years. It contains 943 records with the following features: gender, age, time from diagnosis, body mass index (BMI), glycated hemoglobin (HbA1c), hypertension, and smoking habit. The classification models used were LR, NB, SVM, and RF. The missing data and class unbalance problems were handled using missForest [17], whereas the unbalanced class problem was solved by oversampling the minority class. According to the paper, the maximum accuracy score was reached by LR with 77.7%.

In [18], the authors focused only on studying one complication which is sarcopenia, which is a geriatric syndrome, and it is closely related to the prevalence of type 2 diabetes mellitus (T2DM). The goal of that paper was to make risk assessment of sarcopenia easier by building ML models using SVM and RF. The dataset used in the paper is limited in size with only 132 records of patients aged over 65 and diagnosed with T2DM. It contains several records for each patient, such as age, duration of diabetes, history of hypertension, smoking and drinking habits, as well as some medical records like serum albumin and 25-OH vitamin D3. The missing value problem was solved using a *k*-NN classifier with a *k* set to 10. As mentioned in the paper, the area under the receiver operating characteristic (ROC) curve (AUC) was over 0.7, and the mean AUC of SVM models was higher than that of RF.

Alam et al. [19] studied diabetes-induced nephropathy and cardiovascular disease by building different machine learning algorithms. The dataset used in this paper is a result of a study conducted at the Tokyo Women’s Medical University Hospital and 69 collaborating institutions in Japan. The dataset consists of 779 type 2 diabetes mellitus (T2DM) patients. SMOTE was used to help solve the data-unbalanced problem. Methods such as logistic regression, SVM, naïve Bayes, decision tree, and random forest were used in a supervised environment. RF produced the best results for predicting nephropathy with an AUC score of 0.87.

From the previous literature, it can be noticed that the general research trend is to predict the presence of type 2 diabetes in patients, whereas predicting diabetes complications has received less attention. Moreover, the number of complications studied in most of the available literature is very limited, as it does not exceed two or three complications. Moreover, there is a clear limitation when it comes to the number of features used in each study and the nature of these features. For instance, the number of the available medical tests in [18] is very limited.

Accordingly, one of the objectives of this research was to achieve reliable and improved results in predicting diabetes complications in diabetic patients using various state-of-the-art machine learning algorithms by utilizing a comprehensive UAE-based dataset. An extensive number of experiments was conducted testing several data imputation methods, balancing techniques, as well as studying the effect of applying feature selection to the dataset.

## 3. Materials and Methods

**Figure 1:**
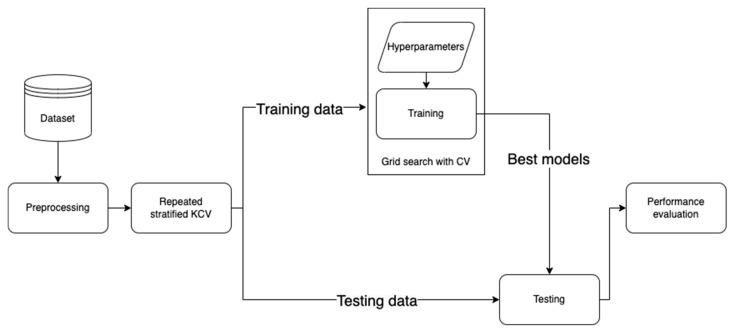
The developed workflow for diabetes complications prediction.

This section elaborates the methodology followed in this work (https://github.com/Yazanjian/Diabetes-Complications-Prediction, accessed on 23 November 2021). Several essential preprocessing steps are discussed along with the machine learning algorithms used. Next, the training process is discussed in detail. Finally, this section presents the evaluation metrics used to assess the learned models’ performance. Figure 1 depicts the workflow of this study.

### 3.1. The Dataset

Utilizing an adequate dataset plays a significant role for any ML problem. In this research, the dataset on hand was collected from the Rashid Centre for Diabetes and Research (RCDR) located in Ajman, UAE [20]. The selection criteria for the collected data must have conformed to the following: all the patients included in this study had already been diagnosed with diabetes and any of its complications under study. Moreover, the dataset mainly consists of medical records which were reported by RCDR.

The dataset consists of 884 patients with 79 input attributes and eight output classes (complications). The input attributes are distributed as follows: 73 numerical attributes and six nominal attributes. From the 73 numerical attributes, we had 64 medical tests, including age, gender, BMI, HbA1c, vitamin D, blood pressure, and diabetes types. For the output (target) attributes, we had the main eight complications, i.e., metabolic syndrome, dyslipidemia, neuropathy, nephropathy, diabetic foot, hypertension, obesity, and retinopathy.

A brief description of these complications is provided below.

Hypertension: according to WHO [21], hypertension—or elevated blood pressure—is a serious medical condition that significantly increases the risks for heart, brain, kidneys and of other diseases. Hypertension occurs when blood pressure is too high.

Obesity: overweight and obesity are defined as abnormal or excessive fat accumulation that may impair health [22]. For adults, WHO defines obesity as having a BMI greater than or equal to 30.

Dyslipidemia is defined as having a high plasma triglyceride concentration, low high-density lipoprotein cholesterol (HDL-C) concentration, and decreased concentration of low-density lipoprotein cholesterol (LDL-C) [23].

Metabolic syndrome is a cluster of metabolic disorders. For example, high blood pressure alone is a serious condition, but when a patient has high blood pressure along with high fasting glucose levels and abdominal obesity, this patient may be diagnosed with metabolic syndrome [24].

Diabetic foot is defined as the foot of diabetic patients with ulceration, infection, and/or destruction of the deep tissues, associated with neurological abnormalities and various degrees of peripheral vascular disease in the lower limb [25].

Neuropathy: nerve damage from diabetes is called diabetic neuropathy. According to CDC [26], high blood sugar can lead to this nerve damage.

Nephropathy is a disease of the kidneys caused by damage to small blood vessels or to the units in the kidneys that clean the blood. People who have had diabetes for a long time may develop nephropathy [27].

Retinopathy is any damage to the retina of the eyes, which may cause vision impairment. Diabetic retinopathy (DR) occurs when high blood sugar damages the blood vessels below the retina [28].

### 3.2. Preprocessing

The given dataset presents issues that require several preprocessing steps that are critical to properly train the machine learning models and fine-tune their performance.

#### 3.2.1. Data Cleaning

The first step in processing the dataset is cleaning it by removing the unnecessary records and attributes by following a systematic procedure. Firstly, the dataset consists of several categorical values that need to be deleted for confidentiality purposes, i.e., hospital number, episode date, and episode description. Furthermore, the dataset consists of missing values for the diabetes type for some patients, which is a critical information in this research since we studied diabetes complications in diabetic patients. Therefore, all the 26 instances suffering from this problem were removed.

Another necessary step in this study is checking the total number of missing values per record (or patient). By testing different percentages using all the classifiers, it was found that removing all the records with >60% of missing values achieved better performance compared to other experiments where this problem was ignored.

Following the approach in [19], the missing values were also investigated per attribute. Based on several experiments, a threshold of 40% was set for this step, meaning that any attribute with missing values larger than or equal to 40% should be dropped from the dataset. Since this dataset has a large number of numerical attributes, it was found that 16 numerical attributes have missing values of more than 40%. More precisely, most of these attributes have more than 90% missing values. This specific threshold was selected experimentally and influenced by the literature [19].

#### 3.2.2. Data Imputation

Handling missing values is essential in training classifiers since most of the available machine learning algorithms cannot be utilized with missing data. For the categorical values available in our dataset, such issues occur only with the nationality attribute. The most frequent value in that column (United Arab Emirates) was thus used to fill the missing values.

On the other hand, three different methods were extensively tested and evaluated to solve the missing values problem in numerical attributes. The first method used to overcome this challenge is by using the mean substitution method [9]. Mean substitution basically is a statistical way to represent and fill any missing value in an attribute (feature) with the average of observed data for that attribute in other records or patients. One of the possible drawbacks of utilizing mean substitution is that it may lead to biased results, hence not reflecting the reality.

Another way to fill the missing values is by using a *k*-NN model to impute the missing values [29]. The *k*-NN classifier goal is to find the nearest neighbors of the missing value based on some predefined distance metric. After that, each missing feature is imputed using values from N nearest neighbors that have a value for the feature. The features of the neighbors are averaged uniformly. If a sample has more than one feature missing, then the neighbors for that sample can be different depending on the particular feature being imputed [30]. Following the approach in [18], the number of neighbors selected for this model is *k* = 10.

The third and last method used in this research was MissForest [17]. This method imputes missing values using random forests in an iterative fashion. The first step in this algorithm is selecting the first attribute which has the least number of missing values (candidate column). After the selection, the missing values in the candidate column are filled by the mean of that column. Moreover, the candidate column is then fitted on a random forest model and treated as the output where other columns in the dataset are treated as inputs to this model. After training the random forest model, the missing rows of the candidate column are imputed using the prediction from the fitted random forest. These steps continue to cover all other columns in the dataset.

To evaluate and compare the performance of all the three algorithms, RMSE was calculated as per Equation (1), for all the three methods as follows. The first step was to simulate the missing value problem by choosing a complete subset of the dataset with no missing values. The total number of records in the complete subset was 217 records. After that, the missing values percentage in the original dataset was calculated and utilized to drop random values from each column in the complete dataset. More precisely, the percentage found was 4.4%, resulting in dropping nine records per column in the complete dataset. After building the artificial dataset, the three mentioned methods were used to impute the missing values. As noticed in Table 1, it was found that MissForest [17] results in the minimum RMSE value followed by *k*-NN [29] and mean methods. It is worth mentioning that Table 1 represents RMSE for some randomly selected attributes as well as the total RMSE for all columns.

(1) $$\mathrm{RMSE}\;=\sqrt{\frac{1}{n}\;\overset{n}{\underset{j\;=1}{\sum }}({\;y}_{j}-\;{\hat{y}}_{j}}{)}^{2}$$

**Figure 2:**
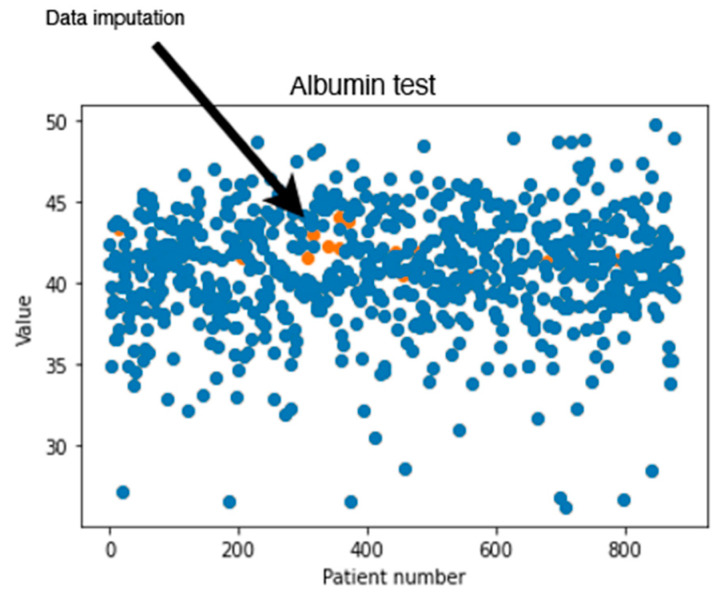
Original and generated albumin test values using MissForest.

In addition to calculating the RMSE values, a visual inspection was performed on the dataset imputed by MissForest. Figure 2 shows an example of the generated values for the albumin test using MissForest. The values in blue represent the values found originally in the dataset, where the orange points show the calculated missing values. It can be noticed that the generated new values are reasonable since they seem to follow the same trend found in the data.

#### 3.2.3. Categorical Encoding

Another needed step is encoding the categorical features in the dataset, which are gender, nationality name, and diabetes type. Encoding is necessary when ML algorithms require numerical data and therefore cannot handle categorical values. For this purpose, one-hot encoding [31] is utilized in this research, that creates a “dummy” variable for each possible category of each nonnumeric feature. Table 2 represents some categorical attributes after applying one-hot encoding.

#### 3.2.4. Data Balancing

**Figure 3:**
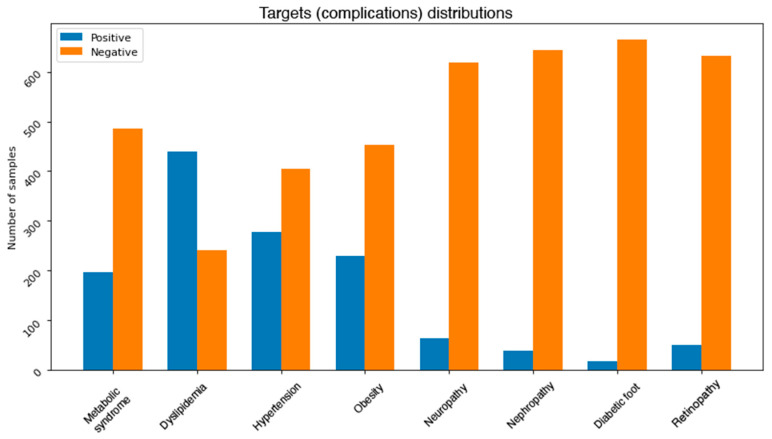
Class distributions for each complication.

One of the common challenges when building and training machine learning and data mining models is dealing with unbalanced dataset. This problem exists in our dataset. Figure 3 depicts the distributions of classes in each complication. It can be noticed that most of the complications are unbalanced. More precisely, neuropathy, nephropathy, retinopathy, and diabetic foot attributes all have some severe unbalanced distributions. For instance, diabetic foot occurs in only 2.5% of the total number of records. This issue needs to be addressed using some effective balancing method. One solution is to reduce the number of instances in the majority class (under sampling) [32], another possible solution is to increase the number of instances in the minority class (oversampling) [33].

**Figure 4:**
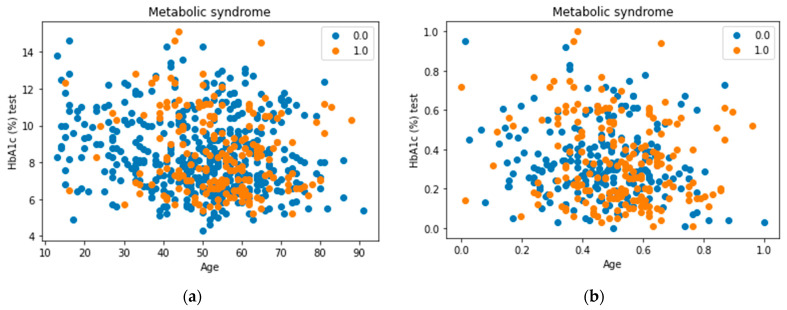
Applying undersampling using cluster centroids. (**a**) Represents the datapoints before applying cluster centroids whereas (**b**) represents the final result of performing undersampling on the dataset.

Several strategies can be followed to perform under sampling on a dataset, and each has some advantages and disadvantages. The first approach tested to balance the dataset in this research is by randomly reducing the number of instances of the majority class, i.e., by removing some samples from the most frequent class based on a given percentage. Despite the simplicity of this method, removing random samples can lead to deleting valuable information which may be preserved in the majority class. To overcome this limitation, cluster centroids are applied [32]. This method undersamples the majority class by replacing a cluster of majority samples with the cluster centroid of a k-means algorithm. This algorithm keeps N majority samples by fitting the k-means algorithm with N clusters to the majority class and using the coordinates of the N cluster centroids as the new majority samples. In addition to experimenting with both methods, a visual inspection of the cluster centroids method was conducted. Figure 4 shows an example of preserving information in the majority class. It can be noticed that most of the dropped datapoints belong to clusters that still have other instances after performing undersampling.

Similarly, several methods can be applied to achieve oversampling. For instance, one method is to duplicate a specific percentage of the minority class. Despite the simplicity of this technique, having duplicates in the dataset will generally not help the model to learn new information. Another approach can be followed by using a proper method such as the synthetic minority oversampling technique (SMOTE) [33]. SMOTE first selects a minority class instance randomly and finds its *k* nearest minority class neighbors. The synthetic instance is then created by choosing one of the *k* nearest neighbors b at random and connecting a and b to form a line segment in the feature space. The synthetic instances are generated as a convex combination of the two chosen instances a and b.

**Figure 5:**
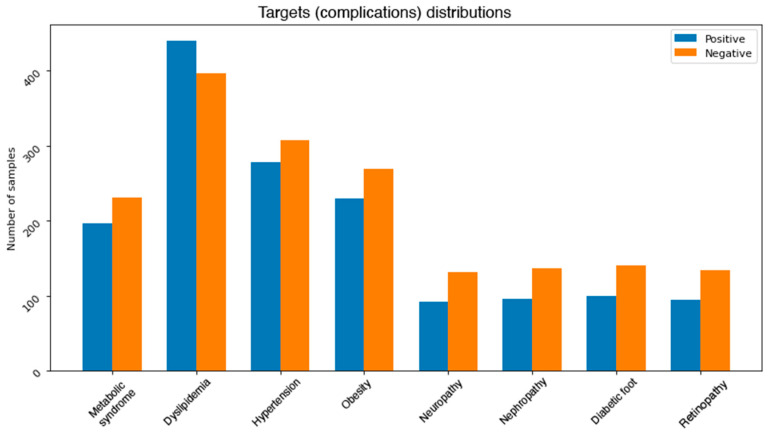
Class distributions for each complication after handling the imbalance problem.

After experimenting with all the previously mentioned balancing methods, a combination of both SMOTE and cluster centroids was used for the final output. Figure 5 shows the final class distributions for all the complications. Since the severity of the imbalance problem varies between the complications, we treated each complication independently.

#### 3.2.5. Data Normalization

As mentioned earlier, most of the attributes available in our dataset are numerical. Moreover, some of these features were recorded with different measurement units. Dealing with such features without any normalization could affect the performance of the models. Therefore, normalization is necessary to rescale all numeric attributes into a range between 0 and 1. Equation (2) describes the normalization formula, where Value is the value needed to be normalized, Max is the maximum value in the column, and Min is the minimum value in the column.

(2) $$\frac{\mathrm{Value}-\mathrm{Min}}{\mathrm{Max}}$$

### 3.3. Machine Learning Models

Several ML learning models were trained to classify the eight complications, namely, logistic regression, SVM, decision tree (CART), random forest, AdaBoost, and XGBoost. These algorithms were selected taking into consideration multiple factors such as the simplicity found in using logistic regression classifiers. LR sometimes surprisingly performs better than other more complicated algorithms, which makes it attractive to apply to this dataset. Equation (3) represents the general formula of LR, where p(X) is the dependent variable, X is the independent variable, $\beta_{0}$ is the intercept, and $\beta_{1}$ is the slope coefficient. The algorithm calculates the probability of the target class by utilizing a simple yet effective linear equation. By using an intercept and slope coefficients for the features in the dataset, the probability is calculated [19]. Although the assumption of linearity between the dependent variable and the independent variables may not be correct in all cases, the simplicity and proven effectiveness of logistic regression make it an attractive algorithm to test in this study [11,16].

(3) $$\log\left(\frac{p\left(X\right)}{1-p\left(X\right)}\right)={\;\beta }_{0}+{\;\beta }_{1}X$$

The second algorithm used is SVM. It is a supervised algorithm used for both regression and classification problems. The objective of the SVM classifier is to find a hyperplane in an N-dimensional space (N—the number of features) that distinctly classifies the datapoints by deciding on which side they fall around the plane [34].

The third algorithm used is the CART decision tree, where classification relies on different nodes and branches starting from top (root node) down to leaves (decisions). The root in the case of the decision tree represents the feature that is used to split the dataset first. This model can be utilized to enhance the understanding of each of the diabetes complications by visualizing the model tree which gives clear and easy-to-follow information. However, decision trees can be prone to overfitting as well as being unstable since adding a new attribute may result in a totally new tree (variance). These challenges can be addressed by tuning different hyperparameters such as the depth of the tree or the number of samples allowed per branch [13]. The criteria to select the attribute to split the data in each of these nodes depend on two measurements, entropy and information gain. Entropy is a measure of disorder or uncertainty and the goal of machine learning models in general is to reduce uncertainty. Information gain, on the other hand, is calculated by comparing the entropy of the dataset before and after a transformation [14]. Equations (4) and (5) can be used to calculate entropy and information gain, respectively.

(4) $$\mathrm{Entropy}\left(S\right)=\underset{i\in I}{\sum }-p_{i}\log_{2}p_{i}$$

where S—the current dataset for which entropy is being calculated, I—the set of classes in S, $p_{i}$—the proportion of the number of elements in class i to the number of elements in set S.

(5) $$\mathrm{Gain}\left(S,\;A\right)=\;E\left(S\right)-\underset{t\in T}{\sum }p_{i}E\left(t\right)$$

where E(S)—entropy of set S, T—the subsets created from splitting set S by attribute A such that $S\;=\cup_{t\in T}t$, $p_{t}$—the proportion of the number of elements in t to the number of elements in set S, E(t)—entropy of subset t.

In addition to the three algorithms explained above, three ensemble algorithms were trained and evaluated in this study. Ensemble approaches which use multiple learning algorithms such as decision trees have proven to be an effective way of improving classification accuracy [35]. On the one hand, bagging methods such as the random forest (RF) algorithm [36] apply the principle of majority voting to the results from several decision trees. On the other hand, boosting algorithms such as AdaBoost [37] and XGBoost [38] are built sequentially by minimizing the errors from previous models while increasing or boosting the influence of high-performance models [35].

### 3.4. Model Training

After processing the dataset and selecting the machine learning algorithms to be used, the next step was to build the actual models by training each algorithm using the processed dataset. Extensive experiments were conducted both to train and fine-tune the models. The rest of this section will discuss the detailed steps.

#### 3.4.1. Cross-Validation

**Figure 6:**
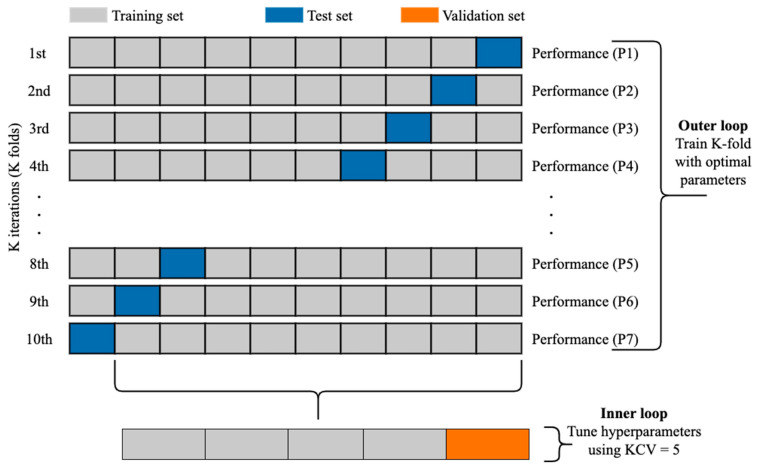
The use of KCV for both hyperparameters tuning and training [9].

The k-fold cross-validation (KCV) technique is one of the most widely used approaches to select a classifier and evaluate its performance [39]. Figure 6 shows the detailed pictorial presentation of data splitting using this technique (with tenfold cross-validation). The dataset was split into K folds. The K − 1 folds were used to train and fine-tune the hyperparameters in the inner loop where the grid search algorithm [40] was employed. In the outer loop, the best hyperparameters and the test data were used to evaluate the model. Since the dataset contains imbalanced records, stratified KCV [41] was used to preserve the percentage of samples for each class the same as in the original percentage. Moreover, for a better evaluation, this process was repeated 10 times. The final performance metric was estimated using Equation (6) where M is the final performance metric for the classifier and $P_{n}$ ∈ R, n = 1, 2, …, K is the performance metric for each fold.

(6) $$M\;=\frac{1}{K}\times \overset{K}{\underset{n\;=1}{\sum }}P_{n}\;$$

#### 3.4.2. Feature Selection

After training the models using the best hyperparameters’ combinations found by grid search, feature selection techniques were applied to the dataset to select the top *N* features for each model. Feature selection played a significant role in this study because the dataset had more than 70 attributes and it was essential to reduce their number and improve the overall performance of the learning models. To this aim, each model built using the complete attribute set was utilized to calculate and select the top five and ten attributes that contributed most to the results. A performance comparison was then conducted to study the effect of utilizing all the features as well as utilizing the selected ones to build several ML classifiers.

In this research, the selection of the top features relied on utilizing the parameters and equations built in each model. As mentioned before, this study used two types of models. The first one was linear models, such as logistic regression and linear SVM, which rely on a linear equation to calculate the final output (or decision). For such estimators, the coefficients of the equations were used to determine the top features to select. The second type of models was tree-based models. For this type, feature importance is calculated as the decrease in node impurity weighted by the probability of reaching that node [42]. The node probability can be calculated by the number of samples that reach the node divided by the total number of samples. The higher the value, the more important the feature.

### 3.5. Evaluation Metrics

To test the performance of the built models, several evaluation metrics were utilized. The first metric used is classification accuracy, which is defined as the percentage of instances classified as their true class labels [16]. Although it is one of the most used evaluation metrics, it does not accurately describe the model performance in case of unbalanced datasets. Hence, it is important to use other techniques in this case. The accuracy of a classifier can be computed using Equation (7), where TP is true positive, TN is true negative, FP is false positive, FN is false negative.

(7) $$\mathrm{Accuracy}\;=\frac{\mathrm{TP}+\mathrm{TN}}{\mathrm{TP}+\mathrm{TN}+\mathrm{FP}+\mathrm{FN}}\times 100\%$$

Other interesting metrics to use are precision and recall. Precision is the percentage of instances that were classified as X and are actually X, whereas recall is defined as the percentage of instances that are actually X and were predicted as X by the classifier [16]. Equations (8) and (9) can be utilized to calculate the precision and recall, respectively.

(8) $$\mathrm{Precision}\;=\frac{\mathrm{TP}}{\mathrm{TP}+\mathrm{FP}}$$

(9) $$\mathrm{Recall}\;=\frac{\mathrm{TP}}{\mathrm{TP}+\mathrm{FN}}$$

The fourth metric is F1-score, which is the harmonic mean of precision and recall. Hence, F1-score is maximum at the value of 1 and minimum at the value of 0 [43]. Equation (10) can be used to calculate F1-score.

(10) $$F1-\mathrm{score}=\frac{2\times \mathrm{Precision}\times \mathrm{Recall}}{\mathrm{Precision}+\mathrm{Recall}}$$

According to what was mentioned earlier, accuracy and F1-score are reported for all the conducted experiments.

## 4. Results

Since we have eight complications in the dataset and because a patient can suffer from multiple complications at the same time, we decided to build binary classifiers for each complication utilizing all the algorithms mentioned before. Moreover, to assess the performance and the effect of training all the algorithms, some baselines should be constructed and compared to the final performance. For that, we established some simple classifiers to compare them with the trained models. The job for each classifier is simply to predict all the instances as the majority class. After accomplishing this step, the accuracy and F1-score were calculated for all these basic estimators. The performance of the baseline classifiers is reported in Table 3 and will be discussed more in the next section.

Table 4 shows the extensive experiments conducted. Since we used k-fold cross-validation (k = 5) for hyperparameters tuning as well as repeated k-fold cross-validation for model training (with k = 10) and a total of 10 repetitions, we conducted 10 × 5 × 10 = 500 experiments for each single model. For each complication, three main experiments were conducted applying all ML algorithms mentioned before. The first group of experiments evaluated the models using all the attributes available in the dataset, whereas the second and third experiments utilized only the top ten and top five attributes, respectively.

## 5. Discussion

The reason behind establishing baseline predictors was that the dataset in hand was used for the first time in this research, and there were no prior performance scores to compare against. By comparing the results in Table 3 with the best results achieved for complications’ models, it can be easily noticed that the final trained models overperformed the performance of the basic classifiers.

Moreover, by comparing our results with the reported accuracy scores in [16], we can notice that our models achieved more than 10% improvement for predicting retinopathy, nephropathy, as well as neuropathy. Table 5 shows further comparisons between our proposed method and other available studies. The accuracy score was used for the comparison since it is the most utilized evaluation metric in the literature.

From the reported results in Table 4, it can be also observed that RF, AdaBoost, and XGBoost mainly achieved the best performance. This observation reinforces the fact that utilizing tree-based ensemble algorithms is essential in such problems. Moreover, the use of “weak” classifiers to build up the final models helps boosting the final performance results. Linear models, especially logistic regression, also performed well for some complications. This indicates that linearity assumption is indeed correct in some cases. Another observation that can be extracted from the results in Table 4 is that in most cases the best results whether for all attributes or top ten or top five are produced by the same algorithm.

By looking at the performance of the best models in Table 4, we can notice that by using only a small subset of all the attributes available we can still achieve acceptable results. The performance achieved by using the selected features’ sets and the total number of features was compared by calculating the mean and standard deviation of the difference between the accuracy scores. For example, the difference between the accuracy score achieved by using all the attributes and by using only the top 10 attributes was 0.0332 ± 0.021, whereas using the top five attributes resulted in a difference of 0.06 ± 0.032. It can be noticed that the degradation of performance resulted by using selected features in most cases was very small. This observation emphasizes the positive effects of applying features selection on the dataset. Furthermore, reducing the number of attributes by more than 60 features had positive effects of reducing the training and prediction time needed. In addition to that, we utilized the best model found for each complication to identify the dominant features that affect it. Based on this step, we found that total cholesterol, diabetes age, gender, BMI, and blood pressure are the most useful features to predict the complications. Moreover, T2DM, weight, low-density lipoprotein (LDL), high-density lipoprotein (HDL), and microalbumin creatinine ratio were also found to be useful. This observation can help us build more sophisticated models by giving more attention and weight to such features. Moreover, physicians can also benefit from such information by also investigating possible relations between these features.

**Figure 7:**
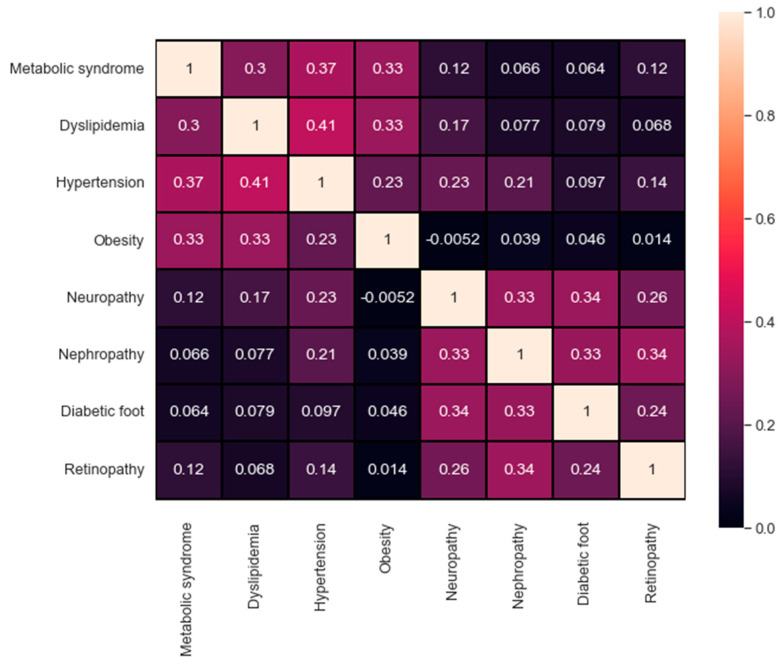
The confusion matrix of the targets’ correlation.

It is also important to study and compare the performance reached for each complication. For instance, one observation was related to the distribution of the output class. The distribution itself plays a significant role and can affect the overall performance. For a better investigation, Figure 7 represents the confusion matrix of the correlation between all the target values available in the dataset. A maximum value of 1 describes the high correlation available, in contrast, a value of 0 indicates no correlation at all. The qualitative and quantitative analysis in Figure 7 demonstrate the correlation between the first four targets (metabolic syndrome, dyslipidemia, hypertension, and obesity) as well as the correlation between the last four targets (neuropathy, nephropathy, diabetic foot, and retinopathy). The correlation in both sets can help explain the findings in Table 4, where the evaluation metrics for each group using all the attributes are indeed adjacent.

**Figure 8:**
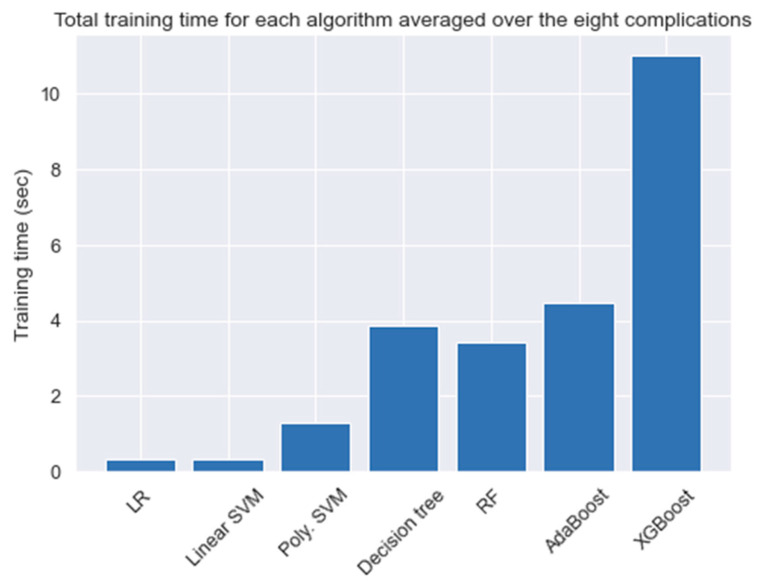
The average time needed to train a model.

For a better understanding of the conducted tests, we calculated the time for each experiment in Table 4. Figure 8 shows the total averaged time needed to train a model using all the attributes based on the algorithm used. Although the training time needed for each algorithm varies slightly over the complications, the general observation is that ensemble methods are found to consume the most amount of training time. This is due to the fact that ensemble algorithms rely on building N numbers of smaller and weaker classifiers to come up with the final output. For this problem, and since the size of the data was relatively small, we neglected the time difference when selecting the best models.

## 6. Conclusions

In this paper, data mining and machine learning algorithms were used to classify and predict eight different diabetes complications. The complications’ set consists of metabolic syndrome, dyslipidemia, hypertension, obesity, diabetic foot, neuropathy, nephropathy, and retinopathy. Furthermore, the dataset used consists of 884 records and 79 attributes. After cleaning the dataset, multiple experiments were conducted to solve the missing value problem. For that, simple mean imputation, *k*-NN as well as MissForest were all tested and evaluated. It was found that MissForest achieved the minimum RMSE score. As a result, it was utilized throughout the rest of this research. After handling all the missing values, one-hot encoding was applied to the categorical attributes such as nationality name, gender, and diabetes type.

Since the dataset on hand suffers from data imbalance issues, different balancing methods were examined. A combination of SMOTE for oversampling the minority class and cluster centroids for undersampling the majority class was used. The algorithms constructed for this study contains logistic regression, SVM, decision tree (CART), random forest, AdaBoost, and XGBoost. Extensive experiments were carried out for model tuning and training. Grid search with cross-validation was employed to select the best hyperparameters for each model. Moreover, *k*-fold cross-validation (KCV) with *k* = 10 was utilized to split the data into training and testing sets. Since the data had imbalanced classes, stratified cross-validation was applied. Moreover, to ensure getting reliable results, the process of CV was repeated 10 times.

Along with using all the attributes to build the models, feature selection was applied to the dataset to select the top ten and five features. The models built using the reduced datasets achieved a comparable performance with the models that utilized all the attributes. Moreover, we utilized this step further for a better understanding of the most dominant features that affect the models’ predictions. Based on our analysis, we observed that total cholesterol, diabetes age, gender, BMI, and blood pressure are the most useful features to predict the complications. Moreover, T2DM, weight, low-density lipoprotein (LDL), high-density lipoprotein (HDL), and microalbumin creatinine ratio were also found to be useful.

## Figures and Tables

**Table 1:** RMSE results for each imputation method.

Method	BMI	Triglycerides	Total RMSE
MissForest	0.6264	1.2051	15.962
*k*-NN	0.9711	1.3514	18.560
Mean substitution	0.8972	1.3378	19.788

**Table 2:** Categorical data before applying one-hot encoding.

Idx	Gender Female	Gender Male	Nationality Name Bahrani	Nationality Name UAE	…	Type 2 Diabetes, Adult Onset	Type 1 Diabetes, Adult Onset
0	0	1	0	1		1	0
1	0	1	0	1		1	0
2	1	0	0	1		0	1
3	1	0	0	1		1	0
4	1	0	0	1		1	0

**Table 3:** Baseline model performance.

Algorithm	Accuracy	F1-Score
Metabolic syndrome	0.5397	0.3505
Dyslipidemia	0.5263	0.3448
Hypertension	0.5256	0.3445
Obesity	0.5402	0.3507
Neuropathy	0.5874	0.3701
Nephropathy	0.5880	0.3703
Diabetic Foot	0.5875	0.3701
Retinopathy	0.5877	0.3702

**Table 4:** Summary of all experiments for the selection of the best-performing classifier for each diabetes complication.

Complication	Algorithms	All Attributes		Top 10		Top 5	
		Accuracy	F1-Score	Accuracy	F1-Score	Accuracy	F1-Score
**Metabolic syndrome**	**LR**	**0.771** ^1^	**0.77**	**0.735**	**0.734**	**0.756**	**0.754**
	SVM Linear	0.763	0.762	0.746	0.744	0.69	0.684
	CART (DT)	0.646	0.639	0.649	0.643	0.651	0.646
	RF	0.753	0.75	0.703	0.7	0.682	0.679
	AdaBoost	0.74	0.738	0.698	0.694	0.673	0.67
	XGBoost	0.738	0.735	0.703	0.7	0.707	0.704
**Dyslipidemia**	LR	0.697	0.677	0.694	0.666	0.695	0.659
	SVM Linear	0.693	0.66	0.685	0.65	0.691	0.654
	CART (DT)	0.649	0.646	0.649	0.646	0.637	0.634
	**RF**	**0.763**	**0.759**	**0.713**	**0.71**	**0.677**	**0.675**
	AdaBoost	0.695	0.692	0.66	0.658	0.621	0.618
	XGBoost	0.747	0.745	0.709	0.706	0.679	0.676
**Hypertension**	LR	0.735	0.732	0.726	0.723	0.702	0.698
	SVM Linear	0.728	0.725	0.725	0.723	0.703	0.699
	CART (DT)	0.678	0.675	0.676	0.673	0.687	0.685
	**RF**	**0.736**	**0.734**	**0.716**	**0.715**	**0.7**	**0.699**
	AdaBoost	0.707	0.705	0.673	0.67	0.607	0.604
	XGBoost	0.725	0.724	0.701	0.699	0.689	0.688
**Obesity**	LR	0.788	0.786	0.775	0.773	0.79	0.788
	SVM Linear	0.793	0.791	0.79	0.788	0.774	0.77
	CART (DT)	0.768	0.765	0.767	0.764	0.768	0.765
	**RF**	**0.8**	**0.799**	**0.803**	**0.802**	**0.78**	**0.779**
	AdaBoost	0.752	0.75	0.739	0.737	0.738	0.736
	XGBoost	0.785	0.784	0.772	0.771	0.767	0.765
**Neuropathy**	LR	0.778	0.764	0.757	0.744	0.708	0.688
	SVM Linear	0.804	0.795	0.786	0.778	0.757	0.744
	CART (DT)	0.704	0.688	0.712	0.697	0.68	0.661
	RF	0.821	0.809	0.783	0.77	0.717	0.701
	AdaBoost	0.811	0.802	0.779	0.769	0.708	0.693
	**XGBoost**	**0.829**	**0.818**	**0.804**	**0.794**	**0.739**	**0.726**
**Nephropathy**	LR	0.825	0.811	0.8	0.784	0.772	0.753
	SVM Linear	0.852	0.844	0.819	0.805	0.822	0.807
	CART (DT)	0.838	0.831	0.838	0.831	0.839	0.831
	RF	0.898	0.896	0.892	0.889	0.891	0.886
	**AdaBoost**	**0.917**	**0.914**	**0.887**	**0.883**	**0.85**	**0.844**
	XGBoost	0.902	0.899	0.885	0.881	0.867	0.861
**Diabetic foot**	LR	0.893	0.888	0.868	0.862	0.843	0.837
	SVM Linear	0.935	0.933	0.908	0.904	0.907	0.903
	CART (DT)	0.86	0.856	0.865	0.86	0.86	0.855
	RF	0.971	0.97	0.944	0.942	0.92	0.916
	AdaBoost	0.941	0.939	0.935	0.933	0.918	0.915
	**XGBoost**	**0.978**	**0.977**	**0.938**	**0.936**	**0.91**	**0.907**
**Retinopathy**	LR	0.796	0.786	0.792	0.779	0.784	0.771
	SVM Linear	0.818	0.812	0.801	0.789	0.792	0.774
	CART (DT)	0.719	0.703	0.722	0.705	0.731	0.717
	RF	0.848	0.842	0.832	0.825	0.801	0.793
	AdaBoost	0.852	0.846	0.828	0.821	0.759	0.748
	**XGBoost**	**0.872**	**0.867**	**0.804**	**0.796**	**0.771**	**0.762**

^1^ Numbers in bold highlight the best classifiers.

**Table 5:** A comparison of recent works developed for predicting diabetes complications using machine learning.

Source	Dataset Size	Best Model	Complication	Accuracy
Our study	884	XGBoost	Diabetic foot	97.8%
[13]	455	ID3	Eye, kidney, heart and diabetic Hyperlipidemia	92.35%
[16]	943	LR	Retinopathy	77.7%
[19]	779	RF	Nephropathy	89%

## Data Availability

The data presented in this study is private.

## Abbreviations

The following abbreviations are used in this manuscript:

DM	Diabetes mellitus
T2DM	Type 2 diabetes mellitus
ML	Machine learning
ID3	Iterative decision tree
*k*-NN	*k*-nearest neighbors
PIDD	PIMA Indians Diabetes Dataset
AUC	Area under the curve
ROC	Receiver operating characteristics
LR	Logistic regression
NB	Naïve Bayes
RF	Random forest
RMSE	Root-mean-square error
RMSEN	Normalized root-mean-square error
RSCV	Repeated stratified cross-validation
KCV	k-fold cross-validation
LDL	Low-density lipoprotein
HDL	High-density lipoprotein
